# Prefabricated CAD/CAM zirconia membrane versus contour augmentation with early implant placement in the anterior maxilla: a randomized controlled clinical trial

**DOI:** 10.1186/s40729-026-00711-4

**Published:** 2026-08-24

**Authors:** Mohammed Mashhout Anas, Amr Mostafa Gabr, Yasser El-Makaky, Mohamed H. Helal

**Affiliations:** Faculty of Dentistry, Oral Medicine, Periodontology, Oral Diagnosis and Radiology Department, Tanta University, Tanta, Egypt

**Keywords:** Guided bone regeneration, CAD/CAM, Customized zirconia membrane, Collagen membrane, Early implant placement, Horizontal ridge augmentation, Anterior maxilla, Randomized controlled trial

## Abstract

**Background:**

Horizontal ridge deficiencies in the anterior maxilla frequently compromise ideal implant positioning and esthetic outcomes. This randomized controlled clinical trial evaluated the clinical performance of customized CAD/CAM zirconia membranes compared with conventional collagen membranes for guided bone regeneration (GBR) performed simultaneously with early implant placement.

**Methods:**

Twenty systemically healthy patients requiring single-tooth replacement in the anterior maxilla underwent early implant placement (4–8 weeks after tooth extraction) combined with simultaneous contour augmentation using a composite graft of autogenous bone and deproteinized bovine bone mineral (1:1). Participants were randomly assigned to receive either a customized CAD/CAM zirconia membrane (test group, *n* = 10) or a double-layer collagen membrane (control group, *n* = 10). The primary outcome was horizontal bone gain (HBG) at 6 months, assessed by standardized CBCT superimposition. Secondary outcomes included soft tissue healing evaluated using the Modified Wound Healing Index (MWHI), membrane exposure (wound dehiscence), and patient-reported postoperative pain assessed using a visual analogue scale (VAS).

**Results:**

All implants survived during the 6-month follow-up period (100% survival rate). Mean HBG was 6.18 ± 0.60 mm in the test group and 5.85 ± 0.37 mm in the control group, with no statistically significant difference between groups (*p* = 0.158). Membrane exposure occurred in all patients receiving customized zirconia membranes (10/10, 100%), whereas no membrane exposure was observed in the collagen membrane group (0/10, 0%). Despite the higher exposure rate, graft stability and horizontal bone regeneration were not adversely affected. Soft tissue healing was significantly more favorable in the collagen membrane group. Postoperative pain decreased significantly over time in both groups, with no significant intergroup differences.

**Conclusions:**

Customized CAD/CAM zirconia membranes achieved horizontal bone augmentation comparable to conventional collagen membranes when used for simultaneous GBR during early implant placement. Although customized zirconia membranes were associated with a substantially higher membrane exposure rate, regenerative outcomes and implant survival remained unaffected, indicating that customized rigid barriers may represent a predictable alternative for horizontal ridge augmentation while offering potential advantages in surgical precision and procedural efficiency.

*Trial registration*: ClinicalTrials.gov NCT06987149.

**Graphical abstract:**

**Figure:**
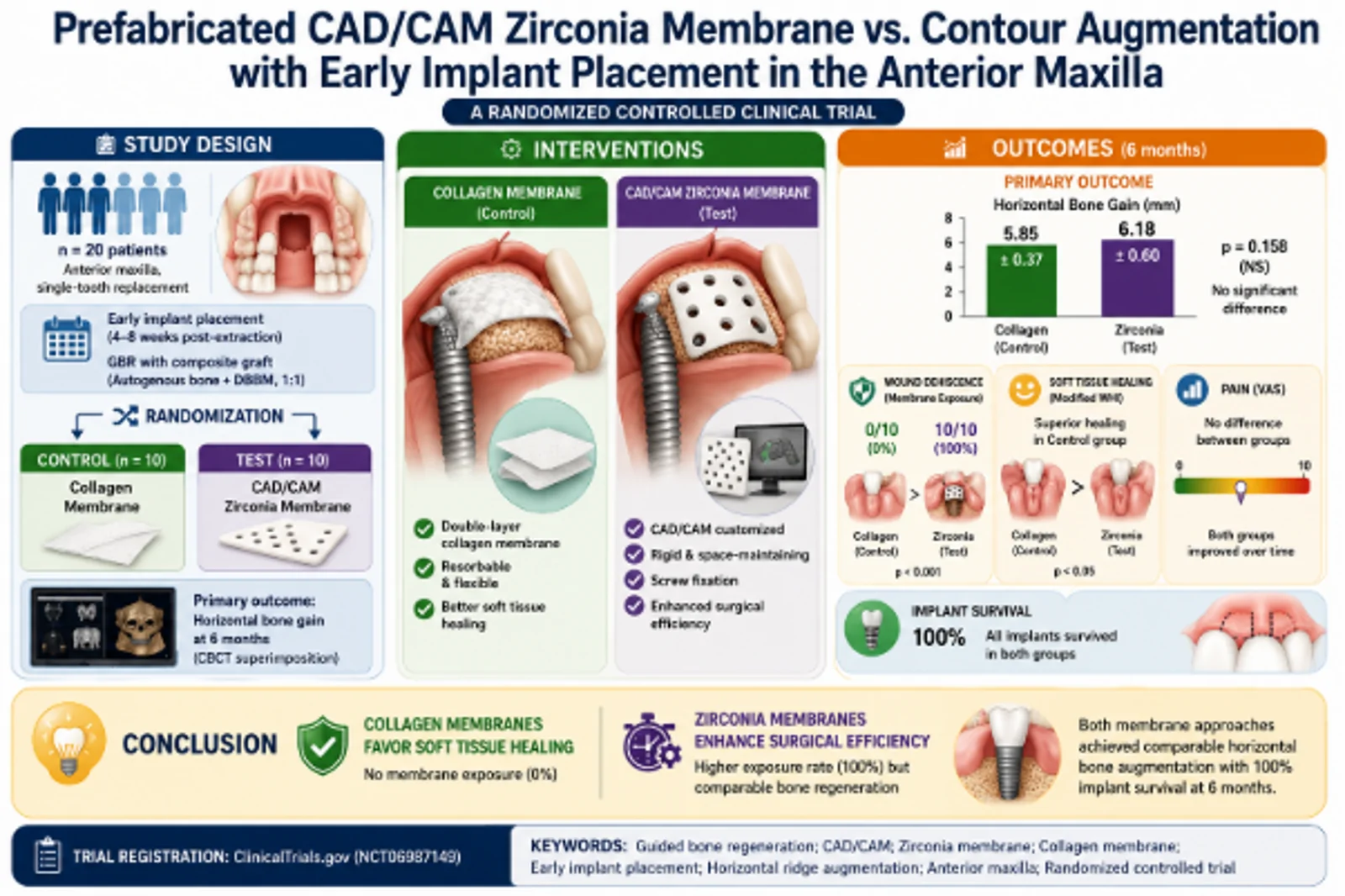

## Introduction

Dental implants are a predictable and widely accepted treatment modality for replacing missing teeth, providing favorable long-term functional and esthetic outcomes. Nevertheless, tooth extraction is invariably followed by progressive alveolar ridge resorption, particularly affecting the buccal bone plate in the anterior maxilla. This dimensional reduction may compromise ideal implant positioning and esthetic rehabilitation. Furthermore, periodontal disease, trauma, and endodontic pathology may aggravate alveolar bone deficiencies, frequently necessitating bone augmentation procedures either before or concomitant with implant placement [1–3]. Various surgical strategies have been proposed to manage alveolar ridge deficiencies, including staged bone augmentation, short implants, and alternative implant positioning. However, these approaches may lead to unfavorable prosthetic designs, compromised esthetic outcomes, and increased biomechanical risks. As a result, augmentation of deficient alveolar bone remains the preferred approach for achieving optimal implant positioning and ensuring long-term peri-implant tissue stability [4–6]. Guided bone regeneration (GBR) is a well-established and biologically sound technique for augmenting alveolar ridge dimensions. The biological principle of GBR relies on the use of barrier membranes to exclude non-osteogenic soft tissue cells, stabilize the blood clot, and create a protected space that promotes osteogenesis [7–9]. Autogenous bone is widely regarded as the gold standard grafting material due to its osteogenic, osteoinductive, and osteoconductive properties and is often combined with xenogeneic bone substitutes to improve volume stability and reduce graft resorption [10–12]. In the esthetically demanding anterior maxilla, the contour augmentation technique—characterized by early implant placement (4–8 weeks following tooth extraction) combined with simultaneous GBR using a bilayer grafting approach—has demonstrated favorable outcomes in maintaining peri-implant bone thickness and soft tissue contours, with high esthetic predictability [13, 14]. Nevertheless, the success of GBR procedures is highly dependent on the properties of the barrier membrane, particularly its ability to maintain space, resist collapse, and support uneventful soft tissue healing [15]. Both resorbable and non-resorbable membranes have been extensively investigated for GBR applications. Collagen membranes remain the most used resorbable barriers due to their biocompatibility, ease of handling, and favorable integration with the surrounding soft tissues. However, their limited mechanical stability may compromise space maintenance, especially in larger or non-contained defects [16, 17]. In contrast, non-resorbable barriers, such as titanium meshes, provide superior rigidity and space maintenance but are often associated with higher membrane exposure rates and increased surgical complexity [18]. Recent advancements in computer-aided design and computer-aided manufacturing (CAD/CAM) technologies have significantly expanded the scope of digital dentistry and introduced new possibilities within regenerative therapies [19–21]. Currently, cone-beam computed tomography (CBCT) data can be utilized to fabricate personalized, patient-specific barrier membranes through CAD/CAM workflows. This approach allows precise adaptation to defect morphology, reduces intraoperative manipulation, and may enhance surgical efficiency and predictability [22, 23]. More recently, customized CAD/CAM zirconia membranes have emerged as a novel non-resorbable alternative for GBR procedures. These membranes combine high mechanical strength with biocompatibility, reduced bacterial adhesion, and favorable soft tissue responses [24–26]. Preliminary clinical and experimental reports suggest that zirconia membranes are capable of maintaining graft stability even in the presence of early membrane exposure, without adversely affecting bone regeneration outcomes [27, 28]. Despite these promising characteristics, clinical evidence supporting the effectiveness of customized CAD/CAM zirconia membranes remains limited, particularly when compared with established contour augmentation protocols using collagen membranes during early implant placement in the anterior maxilla. Therefore, the present randomized controlled clinical trial aimed to compare the clinical and radiographic outcomes of guided bone regeneration using customized CAD/CAM zirconia membranes versus contour augmentation with collagen membranes, both performed simultaneously with early implant placement in the anterior maxilla. The null hypothesis was that no significant differences would be observed between the two treatment modalities in horizontal bone gain and peri-implant soft tissue healing.

## Materials and methods

### Study design and ethical considerations

This prospective, randomized controlled clinical trial was designed as a two-arm parallel study with a 1:1 allocation ratio. The study was designed, conducted, analyzed, and reported in accordance with the CONSORT 2010 statement [29]. Twenty patients (*n* = 20 implant sites) requiring single-tooth implant placement with simultaneous horizontal ridge augmentation in the anterior maxilla (teeth 15–25) were enrolled. The study was carried out at the Department of Oral Medicine, Periodontology, Oral Diagnosis, and Radiology, Faculty of Dentistry, Tanta University, between November 2024 and August 2025. The research protocol was approved by the Research Ethics Committee, Faculty of Dentistry, Tanta University (Approval code: P-OMPDR 5-23-2). All participants provided written informed consent before enrollment, in accordance with the Declaration of Helsinki and its later amendments. The trial was registered at ClinicalTrials.gov (Identifier: NCT06987149) in May 2025.

### Sample size calculation

Sample size calculation was performed using G*Power software (version 3.1.9.2) [30]. Based on previously reported data comparing ridge augmentation techniques [31]. An effect size of 1.255 was assumed for the primary outcome (horizontal bone gain). With a statistical power of 80% (β = 0.20) and a two-sided significance level of 5% (α = 0.05), a minimum of 9 patients per group was required [32, 33]. To compensate for a potential dropout rate of 10%, the final sample size was increased to 10 patients per group, resulting in a total sample of 20 patients [34].

### Patient selection

Patients were recruited following comprehensive clinical and radiographic examinations, including cone-beam computed tomography (CBCT). Inclusion Criteria: Adults aged 20–50 years, Systemically healthy individuals, Good oral hygiene and stable periodontal condition, Favorable soft tissue profile, and Presence of a Type II socket according to Elian et al. [35] at the time of tooth extraction (compromised buccal bone plate [partial or complete dehiscence] with intact facial soft tissue at its normal level).

**Fig. 1:**
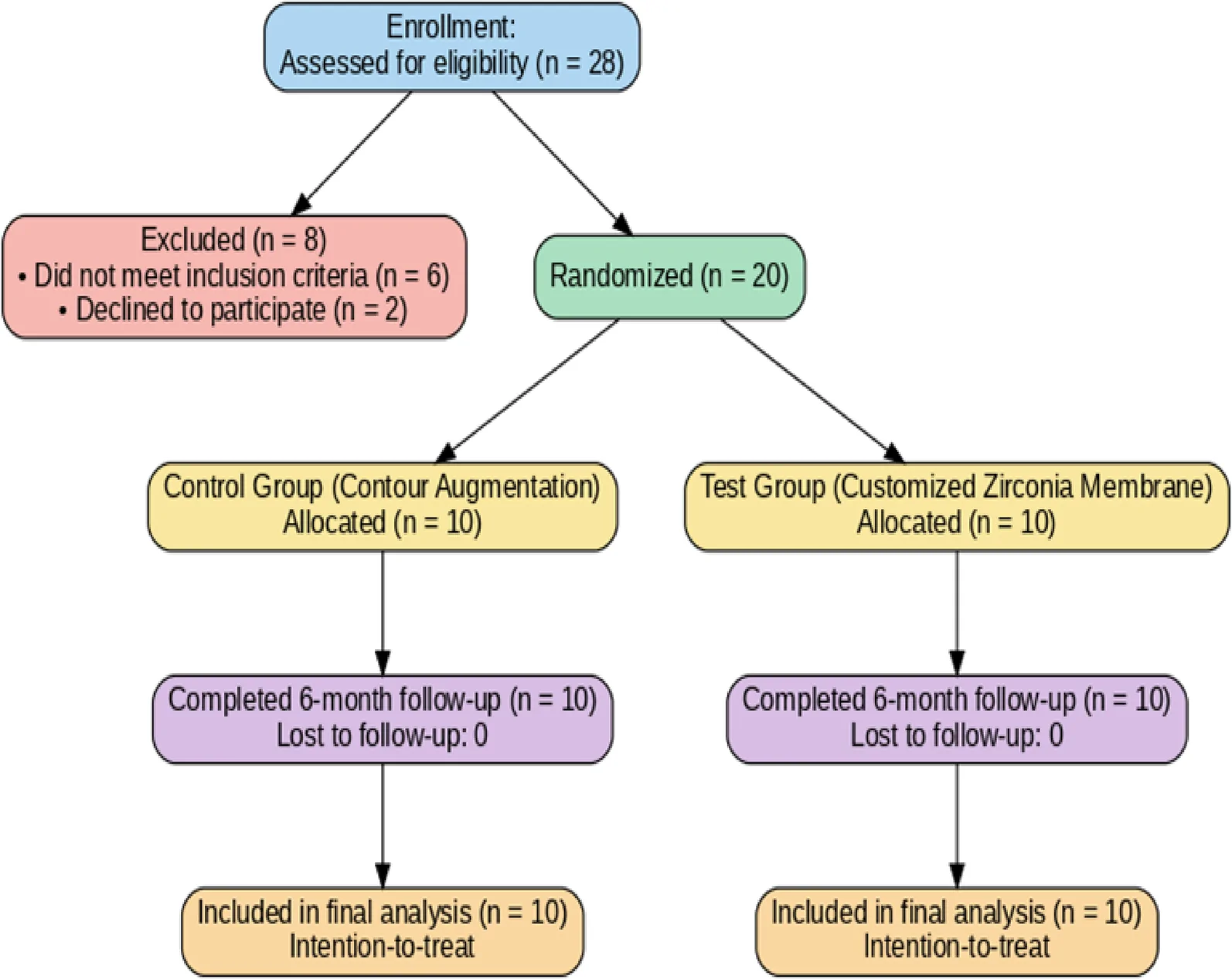
CONSORT flow diagram illustrating the patient recruitment and selection process

Exclusion Criteria: Systemic diseases affecting wound healing or bone metabolism, Uncontrolled diabetes mellitus or metabolic bone disorders, Current smokers, History of head and neck irradiation, Use of medications affecting periodontal health or bone metabolism within the preceding 6 months, Poor oral hygiene or lack of compliance, Active periodontal or periapical infection at the implant site, Pregnant or breastfeeding women, Patient enrollment and allocation are illustrated in the CONSORT flow diagram (Fig. 1).

### Randomization and blinding

Eligible implant sites were randomly allocated into two groups (*n* = 10 per group) using a computer-generated randomization sequence. Allocation concealment was ensured by sealed, opaque, sequentially numbered envelopes prepared by an independent investigator not involved in the surgical procedures. Patients and outcome assessors were blinded to group allocation. Due to the nature of the intervention, the surgeon could not be blinded.

### Intervention groups

Both groups underwent simultaneous implant placement and guided bone regeneration (GBR) using a bilayer grafting approach. The groups differed only in the type of barrier membrane used. Control Group (*n* = 10): GBR using a double-layer collagen membrane combined with a composite bone graft (autogenous bone + deproteinized bovine bone mineral [DBBM], 1:1). Test Group (*n* = 10): GBR using a customized zirconia membrane combined with the same composite bone graft.

### Fabrication of the customized zirconia membrane

**Fig. 2:**
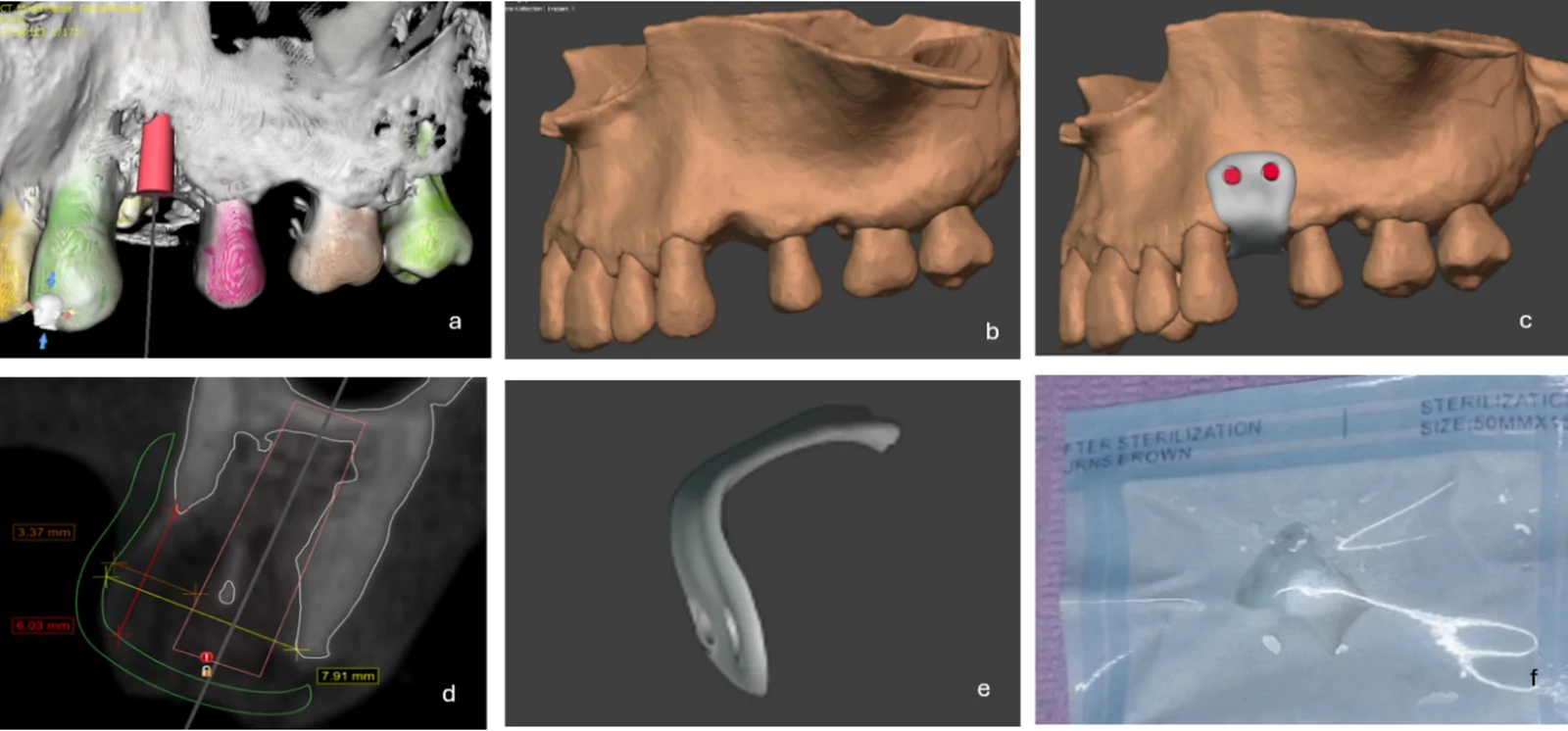
**a**-**f** Digital workflow for customized zirconia membrane fabrication, including STL generation, Blender design, and milling. **a** Implant simulation on BSB; **b** building up the defect and blocking out undercuts on B4D; **c** membrane design over the model on B4D; **d** cross-section showing the membrane, implant, and defect space intended for augmentation; **e** final membrane design; **f** the customized zirconia membrane after sterilization

Preoperative CBCT data were imported into implant planning software to generate a three-dimensional model of the alveolar ridge using the segmentation function in Blue Sky Plan^®^ (Blue Sky Bio LLC, USA). Segmentation of the bony defect was performed, and the defect was digitally augmented using another software, Blender for dental^®^ (TGA, Australia). A customized barrier membrane with a thickness of 0.5–0.7 mm was designed to fully cover the augmented area and extend beyond the defect margins. Fixation screw holes were incorporated based on available bone anatomy. The finalized design was exported as an STL file and milled from zirconia blocks (Ceramill^®^ Zolid HT+; Amann Girrbach, Austria) using a 5-axis CNC milling machine. Following milling, the membrane was sintered according to the manufacturer’s recommendations. The customized zirconia membrane was ultrasonically cleaned in 70% alcohol for 15 min, packaged, and sterilized at 134 °C before surgical application, as outlined by Mandelli et al. [27] (Fig. 2).

### Surgical procedure

**Fig. 3:**
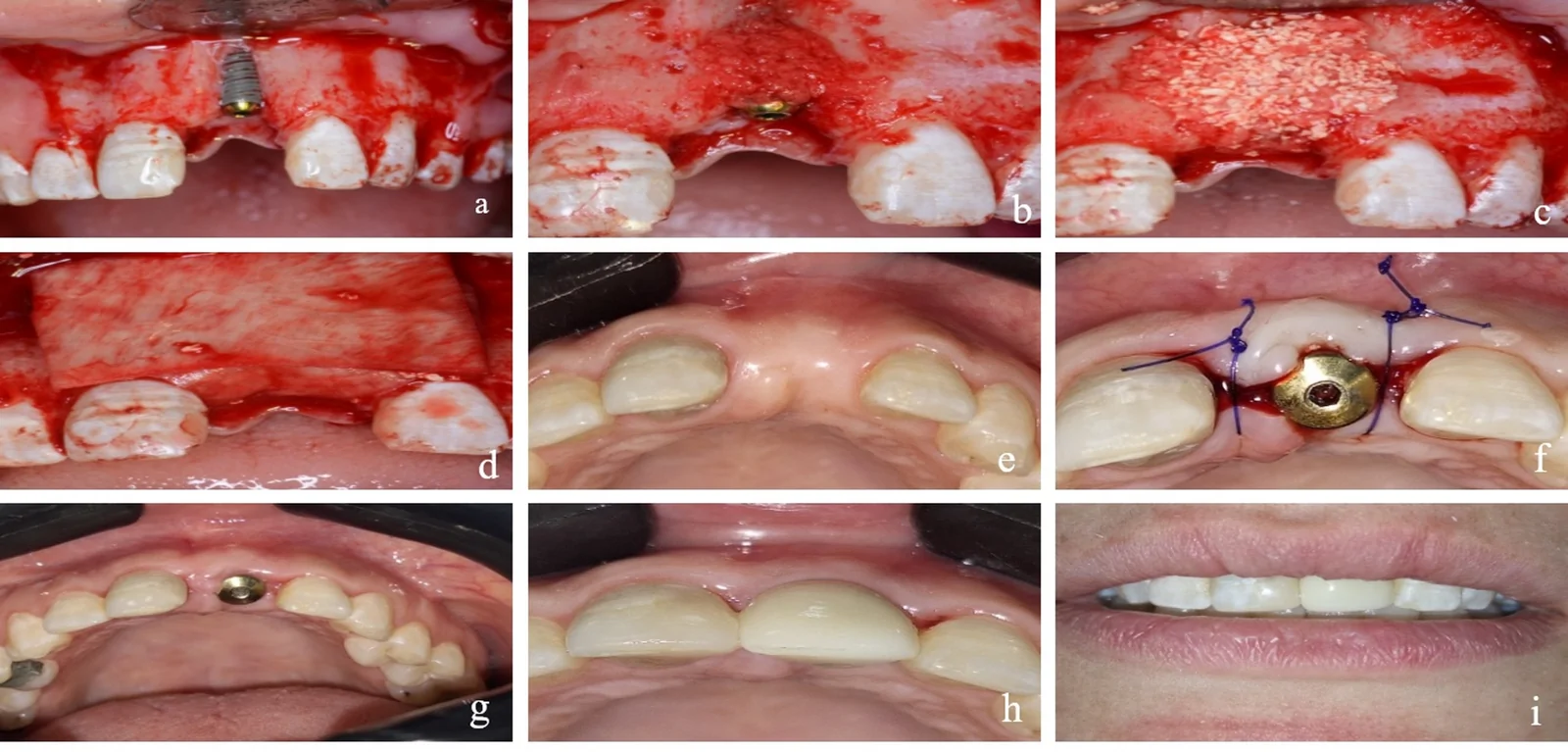
Surgical procedures for the control group. **a** flap reflection with implant placement and bone decortication; **b** autogenous bone chips applied directly to the exposed implant surface; **c** a second layer of composite graft applied to over-contour the ridge; **d** collagen membrane applied using a double-layer technique; **e** follow-up at 6 months; **f** healing abutment and wound closure after second surgery; **g** healing abutment after 2 weeks; **h** final screw retained prothesis; **i** smile line with final prothesis

**Fig. 4:**
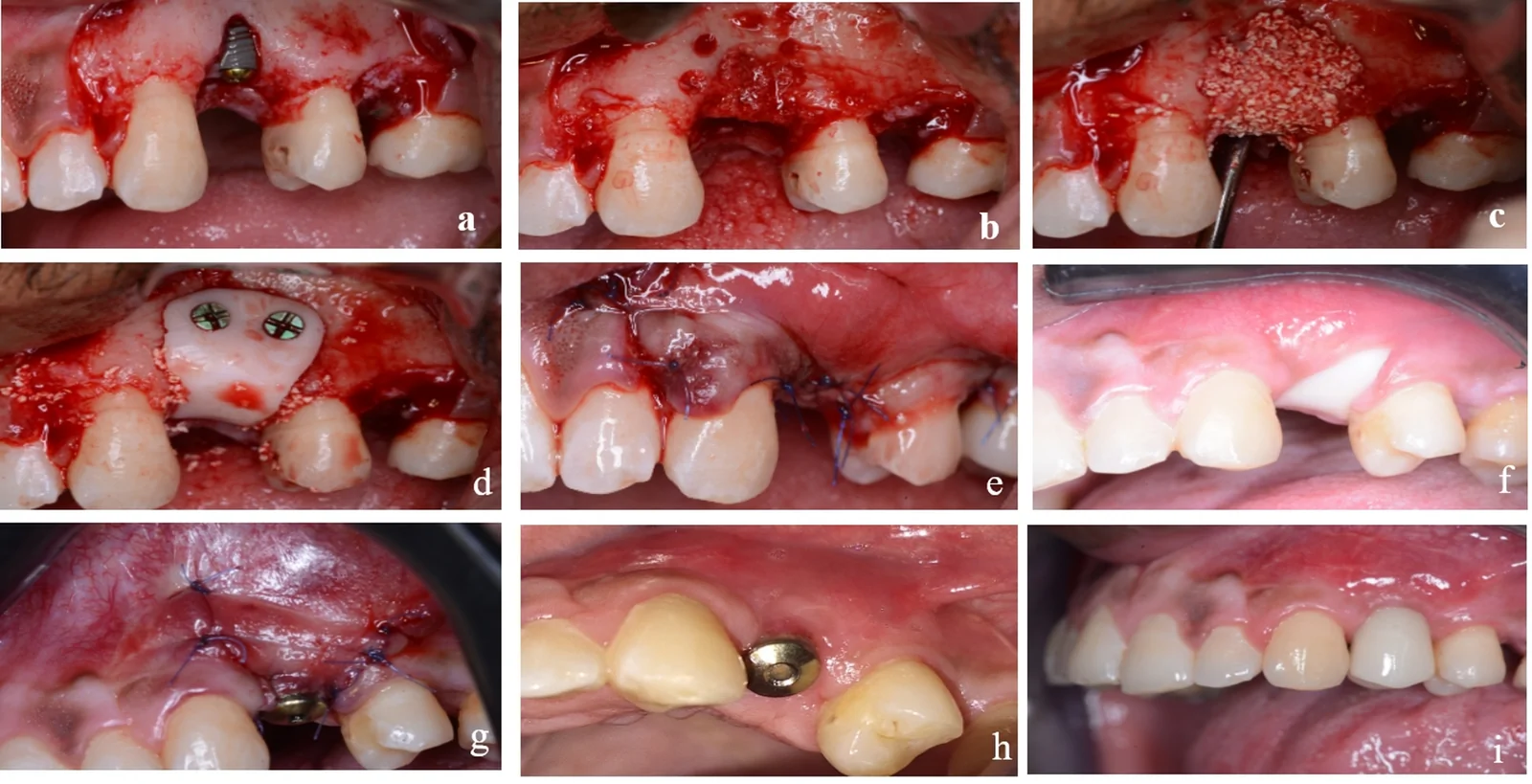
Surgical procedure for the test group (customized CAD/CAM zirconia membrane). **a** Full-thickness flap elevation following a mid-crestal incision with two divergent vertical releasing incisions and implant placement; **b** cortical bone perforations (decortication) and placement of autogenous bone over the exposed implant surface; **c** contour augmentation using a composite graft consisting of autogenous bone and deproteinized bovine bone mineral (1:1); **d** customized CAD/CAM zirconia membrane positioned and stabilized with two fixation screws; **e** immediate primary wound closure with interrupted sutures following customized zirconia membrane fixation; **f** clinical appearance at the 6 month re-entry surgery; **g** removal of the customized zirconia membrane and placement of the healing abutment; **h** peri-implant soft tissue healing 2 weeks after second-stage surgery; and **i** definitive screw-retained implant-supported restoration

All surgical procedures were performed under strict aseptic conditions by the same experienced surgeon. Perioperatively, each patient received one 500/125 mg tablet of amoxicillin/clavulanate potassium every 8 h for 5 days (Hibiotic, Amoun Pharmaceutical Company; Egypt). All procedures were performed under strict aseptic conditions, preceded by a 0.1% chlorhexidine mouth rinse (OROVEX, Sheraton, Egypt) and perioral skin disinfection with 10% povidone-iodine. Local anesthesia was achieved via infiltration at the operative site using Articaine HCl 4% with Epinephrine 1:100,000 (Artinibsa^®^; Inibsa Dental S.L.U., Barcelona, Spain). A full-thick mucoperiosteal flap was elevated using a mid-crestal incision with two apically divergent vertical releasing incisions extending beyond the mucogingival junction. The recipient site was thoroughly debrided, and cortical bone perforations were performed to enhance vascularity. Autogenous bone chips were harvested from the surgical site using a bone scraper and mixed with DBBM (Cerabone^®^, Botiss Biomaterials, Germany) in a 1:1 ratio. Implant osteotomy was prepared according to the manufacturer’s protocol under copious saline irrigation, and implants (BIODEM^®^ (Biodem GmbH, Dusseldorf, Germany) were placed in a prosthetically driven position following established esthetic guidelines. A cover screw was connected after achieving primary stability (insertion torque > 30 N·cm). In the control group, autogenous bone chips harvested from the surgical site using a bone scraper (Helmut Zepf, Germany) were applied directly to the exposed implant surface. A portion of these chips was mixed with DBBM in a 1:1 ratio to prepare the composite graft. A collagen membrane (Lyoplast^®^; Samara State Medical University, Samara, Russia) was adapted and applied using a double-layer technique (Fig. 3). In the test group, the customized zirconia membrane was trial-fitted, positioned over the grafted site, and secured with fixation screws before flap closure (Fig. 4). In both groups, membrane stabilization was further enhanced using a horizontal mattress suture. Periosteal-releasing incisions were performed to achieve tension-free primary wound closure using non-absorbable sutures.

### Postoperative care and follow-up

Postoperative care was standardized across all patients. They were instructed to apply cold packs for the first 24 h, maintain meticulous oral hygiene, and avoid any activities that may inflict trauma on the surgical site for at least 2 weeks. Postoperative medications included chlorhexidine mouth rinse (0.12%) twice daily for two weeks; amoxicillin 875 mg combined with clavulanate 125 mg, administered twice daily for 7 days; and analgesics (Flabu 600 mg, Delta PharmaFactory, Egypt) for 7 days. Follow-up visits were scheduled weekly during the first month and monthly thereafter for 6 months. Sutures were removed 7–14 days postoperatively. CBCT scans were obtained immediately after surgery and at 6 months.

### Second-stage surgery and prosthetic rehabilitation

Six months postoperatively, implants were surgically exposed. In the test group, the zirconia membrane was carefully removed after unscrewing the fixation screws. Healing abutments were placed, and soft tissues were sutured. After 14 days, impressions were taken using the open-tray technique. Provisional restorations were delivered, followed by final screw-retained prostheses after three weeks.

### Outcome measures

#### Primary outcome

**Fig. 5:**
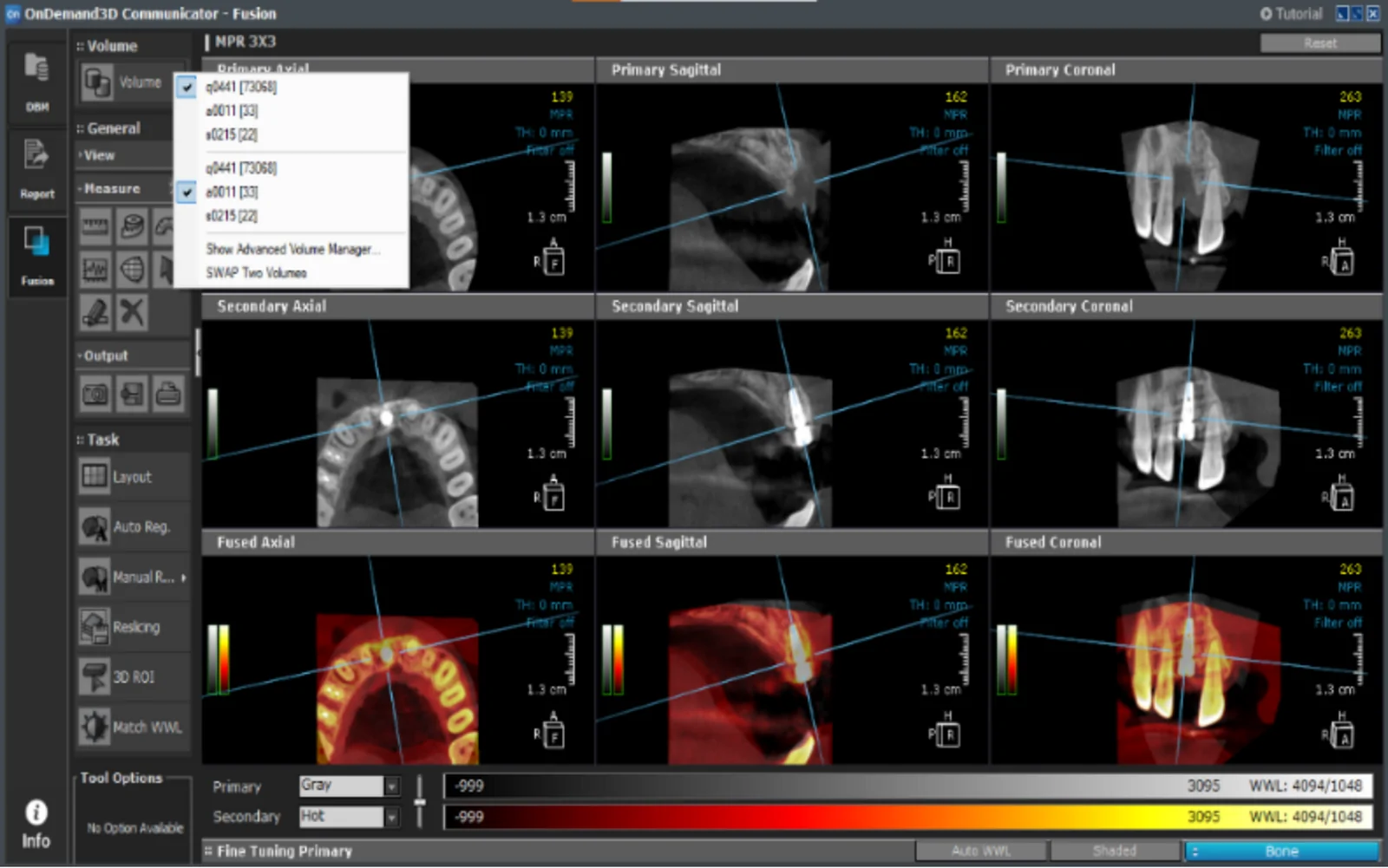
The three fused CBCT datasets

**Fig. 6:**
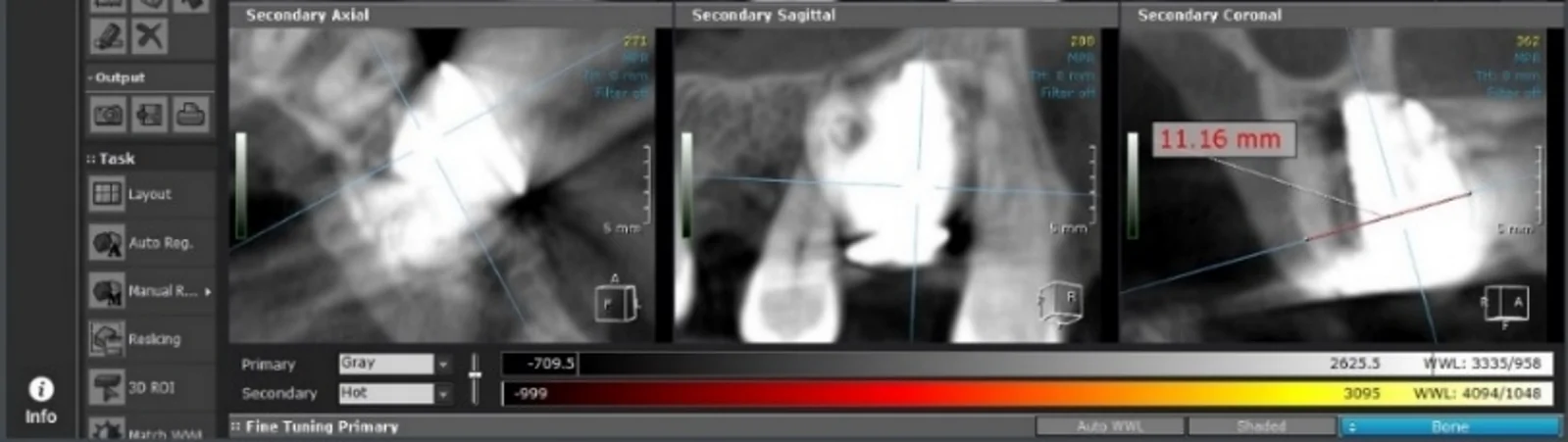
Beam-hardening and partial-volume artifacts associated with the high-radiodensity zirconia membrane used in the test group

CBCT scans were acquired at three time intervals: preoperatively (T0), immediately postoperatively (T1), and 6 months after surgery (T2), using standardized projection parameters for all scans. To enable a consistent and reproducible comparison of peri-implant bone changes between the two augmentation techniques, the preoperative and postoperative datasets were aligned (superimposed) using Fusion registration technology within the Fusion module of OnDemand3D^®^ (Cybermed Inc., Seoul, Korea) (Fig. 5) [36]. Initial alignment was performed manually using anatomical landmarks in the maxilla and palate, followed by refinement with the software’s automated registration tool. Final registration was optimized based on voxel gray-level intensity within the defined anatomical regions. Data from the immediate postoperative scans (T1) were excluded from the final analysis due to significant image distortion caused by beam-hardening and partial-volume artifacts associated with the high-radiodensity zirconia membrane used in the test group (Fig. 6). All radiographic measurements were performed by a single experienced, calibrated, and blinded assessor.

### Horizontal bone gain was assessed radiographically at 6 months post-surgery

**Fig. 7:**
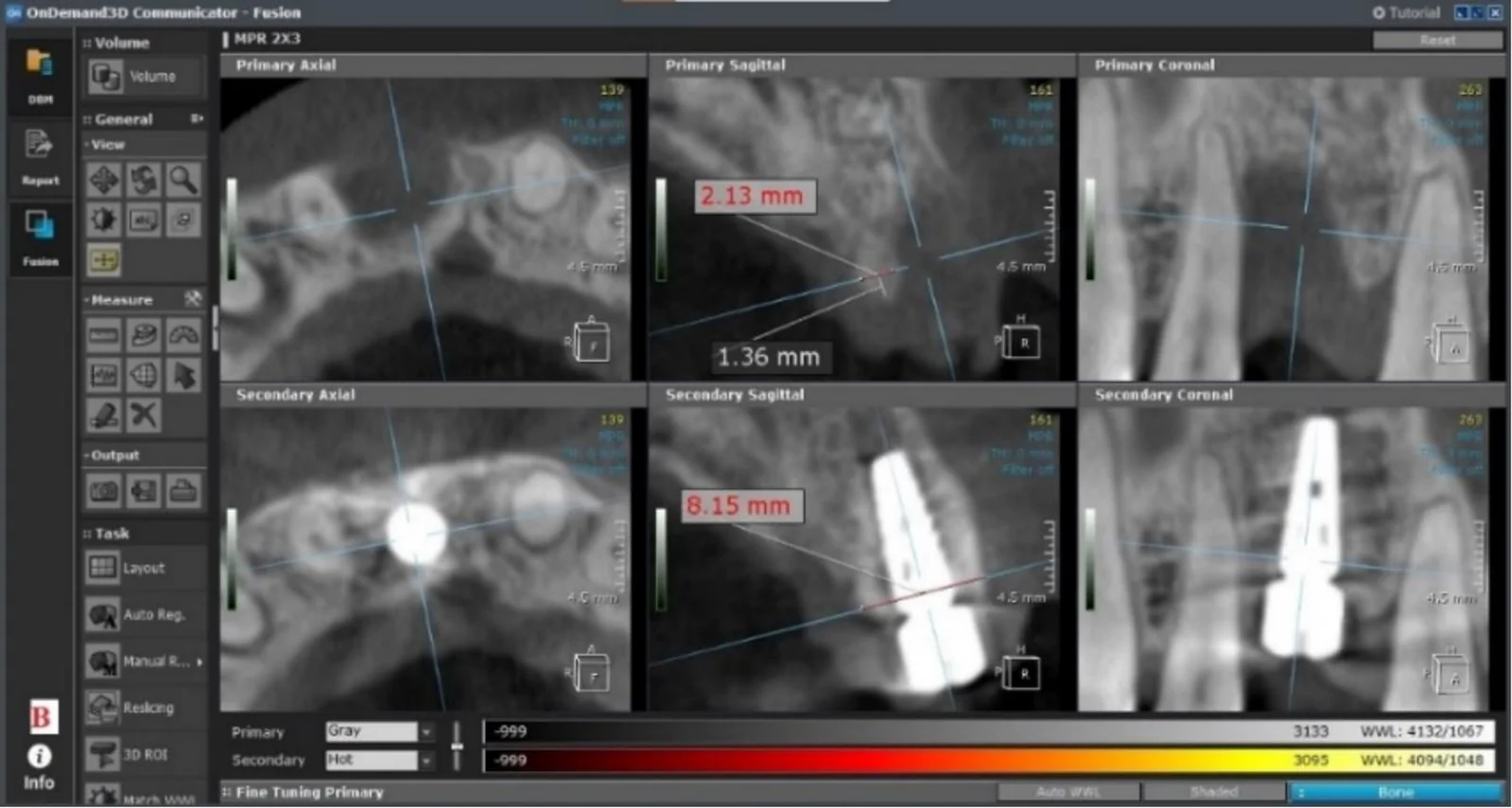
Measurement of pre- and post-ridge width. **a** Reference lines were adjusted on the sagittal view of the primary volume (T0) to serve as guides for subsequent measurements; the axial reference line was positioned approximately 1–1.5 mm apical to the alveolar crest in the primary volume. **b** A linear measurement was superimposed on this axial reference line in the primary volume and subsequently replicated at corresponding anatomical locations in the secondary volumes (T2)

Ridge width (RW) was measured at the same anatomical level across all CBCT scans. Measurements were guided by the software’s reference lines, which were adjusted on the primary (T0) images in alignment with the bone defect and the implant’s long axis. Horizontal ridge width was determined on the sagittal or coronal view according to the implant site, using the axial reference line as a guide, which was placed approximately 1–1.5 mm apical to the palatal alveolar crest in the primary volume. The ruler tool was used to make linear measurements overlaid on the axial reference line in the primary volume and then repeated at corresponding locations on the secondary volumes. Horizontal bone gain (HBG) was calculated by subtracting the initial RW at T0 from that at T2 (HBG = RWT2 − RWT0) (Fig. 7).

### Secondary outcomes

#### Healing process (Modified Wound Healing Index – MWHI)

The quality of soft tissue healing was evaluated using the Modified Wound Healing Index (MWHI) introduced by Huang et al. [37] at baseline, 4, 8, 12, and 24 weeks postoperatively and graded on a 3-point scale: Score 1: Uneventful healing (no/minimal edema/erythema, no suppuration or graft exposure). Score 2: Normal healing (slight–moderate edema/erythema and/or graft exposure, no suppuration). Score 3: Poor healing (pronounced edema/erythema, graft exposure, suppuration).

#### Patient-reported outcome measures (PROMs)

Postoperative discomfort and overall patient satisfaction were assessed using a 100-point visual analog scale (VAS), where 0 indicated “no pain,” and 100 represented the “worst possible pain” [38]. Patients self-reported their levels of discomfort at 1, 6, and 12 h and subsequently on days 1, 3, 7, and 15 following the surgery.

### Statistical analysis

All quantitative data from both primary and secondary outcomes were collected, tabulated, and analyzed using the Statistical Package for the Social Sciences (SPSS, Version 25) [39]. Normally distributed variables were described using minimum, maximum, mean, standard deviation (SD), standard error of the mean (SEM), 95% confidence interval (CI) of the mean, and interquartile range (IQR; 25th–75th percentile) [40]. Non-normally distributed variables were described using minimum, maximum, median, 95% CI of the median, and IQR (25th–75th percentile) [40]. Statistical significance was set at *p*-value < 0.05.

## Results

A total of 28 patients were assessed for eligibility. Eight patients were excluded: six did not meet the inclusion criteria, and two declined to participate (Fig. 1). The remaining twenty patients were randomized equally into two groups: the control group (Contour Augmentation, *n* = 10 implant sites) and the test group (Customized Zirconia Membrane, *n* = 10 implant sites). All randomized patients received the allocated interventions, completed the planned 6-month follow-up, and were included in the final analysis. All implants received definitive screw-retained restorations following successful osseointegration. No prosthetic complications were observed during the study period. Baseline demographic characteristics were comparable between groups (Table 1). No statistically significant differences were observed in sex or age distribution (*p* = 1.00 and *p* = 0.687, respectively). The control group included 6 males and 4 females, with a mean age of 31.40 ± 4.84 years (range: 25–39 years), whereas the test group had an equal sex distribution and a mean age of 32.20 ± 3.85 years (range: 27–38 years) (Table 1).

**Table 1:** Demographic data of the two studied groups

Demographic data	Group		*p*-value
	Control (*n* = 10)	Test (*n* = 10)	
Age (years) Min. – Max. Mean ± SD	25.00–39.00 31.40 ± 4.84	27.00–38.00 32.20 ± 3.85	*p*=0.687 NS
Sex Male (*n* = 11) (55.00%) Female (*n* = 9) (45.00%)	6 (60.00%) 4 (40.00%)	5 (50.00%) 5 (50.00%)	*p* = 1.000 NS

n, number of patients; Min-Max, minimum–maximum; SD., standard deviation; NS, statistically not significant (*p*≥0.05)

### Primary outcome

**Fig. 8:**
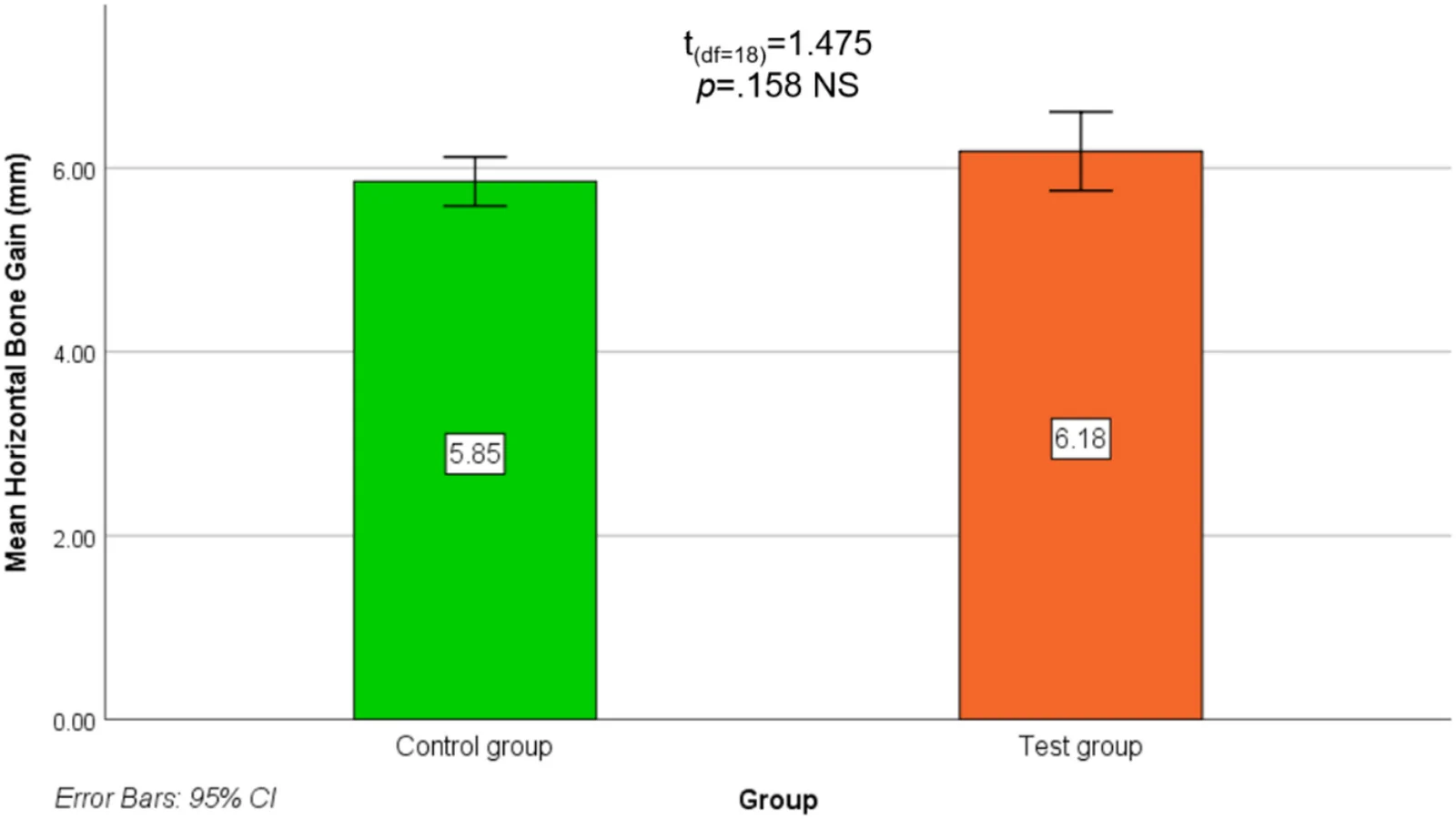
Bar chart of the horizontal bone gain (mm) of the two studied groups

Horizontal bone gain was assessed radiographically at 6 months post-surgery. Horizontal bone gain (HBG) did not differ significantly between groups (*p* = 0.158). In the control group, HBG ranged from 5.10 to 6.49 mm, with a mean ± SD of 5.85 ± 0.37 mm, an SEM of 0.12, a 95% CI of the mean of 5.59–6.12 mm, and an IQR of 5.70–6.08 mm. In the test group, HBG ranged from 5.03 to 6.98 mm, with a mean ± SD of 6.18 ± 0.60 mm, SEM 0.19, 95% CI of the mean 5.75–6.61 mm, and IQR (25th–75th Percentile) of 5.87–6.52 mm (Table 2) and (Fig. 8).

**Table 2:** Horizontal bone gain (mm) of the two studied groups

Horizontal bone gain (mm)	Group		Test of significance *p*-value
	Control (*n* = 10)	Test (*n* = 10)	
Min.–max. Mean ± SD SEM 95% CI of the mean 25th–75th Percentile	5.10–6.49 5.85 ± 0.37 0.12 5.59–6.12 5.70–6.08	5.03–6.98 6.18 ± 0.60 0.19 5.75–6.61 5.87–6.52	t_(df=18)_ = 1.475 *p*=0.158 NS

n, number of patients; Min-Max, minimum–maximum

SD., standard deviation; SEM, standard error of mean

CI, confidence interval; t, independent t-test

df, degree of freedom

NS, Statistically not significant (*p*≥0.05)

### Secondary outcomes

Healing process (Modified Wound Healing Index – MWHI). At baseline, both groups had identical MWHI scores (median = 1.00), with no significant difference (*p* = 1.00). While at 4, 8, 12, and 24 weeks, the test group demonstrated significantly higher MWHI scores (median 2.00) compared with the control group (median 1.00; *p* < 0.001 for all intervals) (Table 3). Membrane exposure was observed in all test-group cases (10/10, 100%) during the healing period. However, no signs of infection or suppuration were detected, and none of the zirconia membranes required premature removal. Intragroup Comparison: In the control group, MWHI remained unchanged throughout follow-up (*p* = NA). In contrast, the test group showed a significant increase in MWHI at all follow-up intervals compared with baseline (*p* < 0.001). Dunn–Šidák multiple comparisons test confirmed higher MWHI scores at all time points compared with baseline (*p* = 0.004 each).

**Table 3:** Modified wound healing index of the two studied groups

Modified healing index	Group		Test of significance *p*-value
	Control (*n* = 10)	Test (*n* = 10)	
Baseline Min.–Max. Median 95% CI of the median 25th–75th percentile	1.00–1.00 1.00 1.00–1.00	1.00–1.00 1.00 1.00–1.00	Z_(MW)_ = 0.000 *p* = 1.000 NS
4 weeks Min.–Max. Median 95% CI of the median 25th–75th percentile	1.00–1.00 1.00 1.00–1.00	2.00–2.00 2.00 2.00–2.00	Z_(MW)_ = 4.359 *p*<0.001*
8 weeks Min.–Max. Median 95% CI of the median 25th–75th percentile	1.00–1.00 1.00 1.00–1.00	2.00–2.00 2.00 2.00–2.00	Z_(MW)_ = 4.359 *p*<0.001*
12 weeks Min.–Max. Median 95% CI of the median 25th–75th percentile	1.00–1.00 1.00 1.00–1.00	2.00–2.00 2.00 2.00–2.00	Z_(MW)_ = 4.359 *p*<0.001*
24 weeks Min.–Max. Median 95% CI of the median 25th–75th percentile	1.00–1.00 1.00 1.00–1.00	2.00–2.00 2.00 2.00–2.00	Z_(MW)_ = 4.359 *p*<0.001*
Friedman test *p*-value	NA	c^2^_(df=4)_ = 40.00 *p*<0.001*	

n, number of patients

Min-Max, minimum–maximum

SD., standard deviation

SEM, standard error of mean

CI, confidence interval

MW, Mann-Whitney U

df, degree of freedom

NA, not applicable

*, Statistically significant (*p*<0.05)

NS, statistically not significant (*p*≥0.05)

### Patient-reported outcome measures (PROMs)

VAS scores showed no significant differences between groups at any postoperative time point. Median VAS scores were comparable at 1 h (20.0; *p* = 0.377), 6 h (30.0; *p* = 0.905), 12 h (30.0; *p* = 0.548), 1 day (10.0; *p* = 1.00), 3 days (0–5.0; *p* = 0.661), 7 days (0.0; *p* = 1.00), and 15 days (0.0; *p* = 1.00) (Table 4).

**Table 4:** VAS of the two studied groups

VAS	Group		Test of significance *p*-value
	Control (*n* = 10)	Test (*n* = 10)	
1 hour Min.–max. Median 95% CI of the median 25th percentile–75th percentile	15.00–20.00 20.00 20.00–20.00 15.00–20.00	15.00–30.00 20.00 20.00–30.00 15.00–25.00	Z_(MW)_ = 0.883 *p*=0.377 NS
6 hours Min.–max. Median 95% CI of the median 25th percentile–75th percentile	25.00–40.00 30.00 30.00–40.00 25.00–40.00	20.00–40.00 30.00 30.00–40.00 25.00–40.00	Z_(MW)_ = 0.120 *p*=0.905 NS
12 hours Min.–max. Median 95% CI of the median 25th percentile–75th percentile	20.00–30.00 30.00 30.00–30.00 25.00–30.00	20.00–40.00 30.00 30.00–40.00 25.00–30.00	Z_(MW)_ = 0.601 *p*=0.548 NS
1 day Min.–max. Median 95% CI of the median 25th percentile–75th percentile	10.00–10.00 10 10.00–10.00	10.00–10.00 10 10.00–10.00	Z_(MW)_ = 0.00 *p* = 1.00 NS
3 days Min.–max. Median 95% CI of the median 25th percentile–75th percentile	0.00–10.00 0 0.00–10.00	0.00–10.00 5 0.00–10.00 0.00–10.00	Z_(MW)_ = 0.438 *p*=0.661 NS
7 days Min.–max. Median 95% CI of the median 25th percentile–75th percentile	0.00–0.00 0.00 0.00–0.00	0.00–0.00 0.00 0.00–0.00	Z_(MW)_ = 0.00 *p* = 1.00 NS
15 days Min.–max. Median 95% CI of the median 25th percentile–75th percentile	0.00–0.00 0.00 0.00–0.00	0.00–0.00 0 0.00–0.00	Z_(MW)_ = 0.00 *p* = 1.00 NS
Friedman test *p*-value	c^2^_(df=6)_ = 57.456 *p*<0.001*	c^2^_(df=6)_ = 56.949 *p*<0.001*	

n, number of patients; Min-Max, minimum–maximum

SD., standard deviation; SEM, standard error of mean

CI, confidence interval; MW, Mann-Whitney U

df, degree of freedom

*, Statistically significant (*p*<0.05); NS, statistically not significant (*p*≥0.05)

Intragroup analysis using the Friedman test revealed a statistically significant decrease in VAS scores over time in the control and test groups (*p* < 0.001 for both). The Dunn–Šidák multiple comparisons test revealed that, in the control group, scores decreased significantly at 7 and 15 days compared with 1 h (*p* = 0.016 for both), at 3, 7, and 15 days compared with 6 h (*p* < 0.001 for all), and at 3, 7, and 15 days compared with 12 h (*p* = 0.004, < 0.001, and < 0.001, respectively). Similarly, in the test group, VAS scores decreased significantly at 7 and 15 days compared with 1 h (*p* = 0.011 for both), at 3, 7, and 15 days compared with 6 h (*p* = 0.005, < 0.001, and < 0.001, respectively), and at 3, 7, and 15 days compared with 12 h (*p* = 0.001, < 0.001, and < 0.001, respectively).

## Discussion

Achieving functional and aesthetic implant therapy in the anterior maxilla is challenging due to ridge deformities and post-extraction remodelling, which compromise implant positioning and prosthetic outcomes [41–43]. In the first weeks after extraction, rapid osteoclastic activity causes ridge resorption, especially buccally at single extraction sites. Although osteoclastic activity subsides by week eight, osteoblastic activity remains elevated, creating a biologically favourable window for regenerative interventions [44–46]. Early implant placement (4–16 weeks post-extraction) takes advantage of this healing timeline, offering more favourable defect configurations, greater keratinized mucosa width, and easier tension-free flap closure. This approach thereby enhances the predictability of regenerative procedures in sites with thin or deficient buccal bones [25, 46, 47].

Guided bone regeneration (GBR) is a well-established approach for ridge augmentation that combines barrier membranes with bone grafts [48–50]. However, the ideal membrane remains debated [26, 51]. Collagen membranes are widely used for their easy handling, resorbability, and low complication rates, but risk graft collapse due to limited rigidity [52]. Non-resorbable rigid membranes (titanium, PTFE) maintain space effectively but require precise, time-consuming intraoperative adaptation, risking instability [26, 53]. Advances in digital dentistry have enabled the fabrication of patient-specific, customized CAD/CAM membranes based on CBCT data, providing an intimate, defect-specific fit that reduces surgical time [27, 54, 55]. Among the available materials, zirconia offers high biocompatibility, favourable fibroblast response, and resistance to microbial colonization, making it a promising alternative to conventional membranes [56, 57].

In this context, the present study evaluated the efficacy of customized (prefabricated CAD/CAM) zirconia membranes (test group) versus contour augmentation using collagen membranes (control group), both performed simultaneously with dental implant placement in the anterior esthetic zone, to determine their impact on bone regeneration, wound healing, and patient-centered outcomes. Both groups received the same combination of autogenous and xenogeneic bone particulates.

In our study, all implants were placed following an early placement protocol (4–8 weeks post-extraction). This approach is supported by Bassir et al. [58], who, in a systematic review and meta-analysis, reported significantly less marginal bone loss with early placement compared with immediate placement, in which greater bone loss is attributed to the inevitable post-extraction alveolar remodelling.

In this study, autogenous cortical bone chips were harvested intraorally from the same surgical site using a sharp bone scraper and applied directly over the exposed implant surface [24, 59]. This is consistent with Chappuis et al. [25], who demonstrated that such chips provide a rich source of viable osteogenic cells, growth factors, and non-collagenous proteins, and viable osteocytes at the critical bone-implant interface, collectively accelerating new bone formation during the early healing phase.

Graft volume stability was further enhanced using a bilayer approach, where a second layer of composite graft consisting of autogenous bone and DBBM in a 1:1 ratio was applied to over-contour the ridge. DBBM exhibits a highly crystalline structure and low resorption rate, contributing to long-term volume stability compared with autografts or allografts [60, 61]. As demonstrated by Galindo-Moreno et al. [62], this 1:1 ratio provides a synergistic effect by combining the biological activity of autogenous bone with the structural stability of DBBM. While autogenous bone undergoes natural turnover, creating a new haversian system that facilitates osteoblast invasion and active bone remodelling, DBBM maintains scaffold integrity due to its slow resorption, thereby preserving the augmented volume over time.

In the test group, customized zirconia membranes were fabricated using a fully digital CAD/CAM workflow based on patient-specific CBCT data [27, 55]. These membranes demonstrated easy handling, precise defect adaptation, and unambiguous positioning, unlike conventional rigid membranes that require extensive intraoperative contouring. However, their inherent brittleness limited flexibility and necessitated careful torque control during fixation [26, 27, 53]. These observations align with Sakr et al. [63], who reported high positional accuracy and minimal deviation between virtual planning and intraoperative placement of digitally designed zirconia membranes.

Removal of the zirconia membranes at second-stage surgery was straightforward, requiring only the unscrewing of fixation screws. This finding is consistent with Mandelli et al. [27], who reported that zirconia meshes exhibited no integration or adhesion with the surrounding soft and hard tissues, thereby allowing rapid and uncomplicated retrieval at the time of re-opening surgery, unlike other barrier materials, whose removal is often delicate and time-consuming. Following membrane removal, a thin periosteum-like soft-tissue layer was consistently observed overlying the regenerated bone. This observation corroborates Malmstrom et al. [55], who reported a 0.5–1.0 mm periosteum-like fibrous tissue layer beneath ceramic membranes, containing capillary blood vessels and showing no inflammatory or adverse tissue response.

In this study, pre- and postoperative CBCT datasets were analyzed using the fusion module of OnDemand3D software to enable precise detection of subtle bone changes, allow reliable comparison of peri-implant volumetric bone changes, and minimize errors associated with patient positioning and imaging variability across CBCT imaging intervals [64]. At 6 months, both groups demonstrated substantial horizontal bone gain (HBG) with no statistically significant intergroup differences, although the test group showed slightly higher HBG values.

The mean HBG in the control group (5.85 ± 0.37 mm) aligns with Gultekin et al. [65], who reported HBG of 5.42 ± 0.76 mm using autogenous bone combined with DBBM (1:1) under a collagen membrane in horizontally deficient maxillary ridges. Our results also fall within the upper range reported by Shan-Huey Yu et al. [66], who observed HBG of 3–5.6 mm using collagen membranes with mixed xenogeneic and autogenous grafts, applied either simultaneously or in a staged approach. In contrast, Saravanan et al. [67] reported markedly lower HBG (~ 1.4 mm) at 2, 4, and 6 mm apical to the crest in Seibert’s Class I maxillary defects treated with a similar graft-membrane combination.

In the test group, mean HBG (6.18 ± 0.60 mm) corresponds with Heikal et al. [68], who observed greater HBG when zirconia sheets were combined with xenografts compared to zirconia alone (6.86 ± 1.00 mm vs. 5.76 ± 1.18 mm). This underscores the synergistic effect of rigid membranes with osteoconductive grafts, reflected in our protocol pairing zirconia membranes with autogenous and DBBM particles. Similarly, Albash et al. [69] reported HBG of 5.55 mm using customized zirconia membranes with particulate allogeneic grafts in the anterior maxilla. While Souidan et al. [70] reported a lower mean HBG (2.18 ± 1.05 mm).

It should be emphasized that the current body of evidence on zirconia membranes remains limited and heterogeneous, primarily derived from case reports and small case series with short follow-up. Uriarte et al. [71].

In this study, soft tissue healing was assessed using the Modified Wound Healing Index (MWHI), revealing a significant difference between groups. The control group consistently maintained a score of 1 throughout the follow-up, indicating uneventful healing. In contrast, the test group exhibited a significant increase to a score of 2 from the fourth week onward, persisting through 24 weeks. This increase reflects premature zirconia membrane exposure; however, no clinical signs of suppuration, edema, erythema, or acute inflammation were observed. The uneventful healing observed in the control group aligns with Keddar et al. [73] and Gultekin et al. [65], both of whom reported no mucosal complications such as dehiscence, membrane exposure, or infection when collagen membranes were combined with particulate bone grafts. Conversely, Meloni et al. [17] noted early collagen membrane exposure in 3 of 18 patients undergoing GBR with native collagen membranes and a 1:1 mixture of xenograft and autogenous bone.

Membrane exposure in the test group closely parallels Souidan et al. [70], who reported a 90% exposure rate (9 of 10 cases). Although one case in their study experienced partial graft loss, most healed uneventfully without complete graft failures, consistent with the present findings, where exposure did not compromise regenerative outcomes. Similarly, Melek et al. [74] documented membrane exposure in three out of seven cases. In our study, exposed membranes were managed in accordance with Mandelli et al. [27], whereby patients were instructed to maintain meticulous local hygiene and use antiseptic mouth rinses. Notably, no signs of infection were observed throughout the healing process. By contrast, Malmström et al. [55] reported excellent soft tissue responses with no zirconia membrane exposure, while Mandelli et al. [27] observed premature exposure in only one of nine cases. Variations in exposure rates across studies likely reflect anatomical and surgical variables. In the current trial, the limited augmentation spans restricted flap mobility, and adjacent teeth may have hindered tension-free closure. Moreover, strong perioral muscle activity and frequent lip movement in the maxillary anterior region exert continuous pulling on the flap margins, predisposing to dehiscence [75].

Patient-reported outcomes for postoperative discomfort and overall satisfaction were assessed using a 100-point visual analog scale (VAS). No significant differences were observed between the two groups at any time point. Intragroup analysis showed a significant reduction over time, with both groups experiencing a modest pain peak at 6–12 h (median 30) that declined steadily, becoming negligible by day 3 and resolving by day 7. Notably, VAS scores in the range of 30–54 were classified as moderate pain [76]. These findings indicate that both collagen and zirconia membranes are clinically acceptable from a patient-centered perspective, as neither imposed additional discomfort beyond the normal postoperative course. These findings are consistent with those of Urban et al. [77], who reported that postoperative discomfort following immediate implant placement with three regenerative techniques was generally mild to moderate, peaking around 5–6 h and declining sharply by day 3, with no significant differences between interventions.

Our findings reflect the expected healing trajectory and underscore the transient, self-limiting nature of pain in regenerative procedures under proper postoperative management. Also, suggest that pain after GBR is more closely associated with soft tissue manipulation, surgical trauma, flap design, and patient-specific healing responses than with membrane type [78, 79]. Similarly, Dawar et al. [80] confirmed that short-term PROMs such as pain, swelling, and restricted mouth opening after GBR with resorbable membranes are strongly influenced by flap design, surgical handling, and soft tissue characteristics, all of which significantly influenced the severity of symptoms and, consequently, the postoperative quality of life.

The current study was limited by its small sample size and short follow-up, single-center and single-operator design, lack of histological evaluation, and absence of assessment of zirconia membrane retrieval morbidity or cost. Overall, the present study supports the reliability of simultaneous guided bone regeneration with early implant placement, demonstrating comparable clinical, radiographic, and patient-reported outcomes for both collagen and customized zirconia membranes. While minor variations were observed, these did not translate into clinically meaningful differences, underscoring that membrane type alone may not be the decisive factor in short-term healing success. Instead, careful surgical execution, flap design, and grafting protocol appear to play a more influential role. Within the limitations of the present study, these results highlight the clinical applicability of both membranes and provide a foundation for considering material selection in relation to specific defect morphologies, patient factors, and long-term stability. Notably, collagen membranes were associated with more favorable soft tissue healing, whereas zirconia membranes offered superior intraoperative efficiency.

## Conclusions

Both membrane approaches provided comparable horizontal bone augmentation when combined with early implant placement. Collagen membranes favored optimal soft-tissue healing, whereas customized zirconia membranes improved surgical efficiency without negatively affecting bone regeneration, despite demonstrating higher membrane exposure rates.

## Data Availability

The datasets generated and analyzed during the current study are available from the corresponding author upon reasonable request.

## References

[1] Moraschini V, et al. Evaluation of survival and success rates of dental implants reported in longitudinal studies with a follow-up period of at least 10 years: a systematic review. Int J Oral Maxillofac Surg. 2015;44(3):377–88. 25467739 10.1016/j.ijom.2014.10.023

[2] Jivraj S, Chee W. Rationale for dental implants. Br Dent J. 2006;200(12):661–5. 16799436 10.1038/sj.bdj.4813718

[3] Khalifa AK, et al. To what extent residual alveolar ridge can be preserved by implant? A systematic review. Int J Implant Dent. 2016;2(1):22. 27878769 10.1186/s40729-016-0057-z PMC5120622

[4] Chiapasco M, Zaniboni M, Boisco M. Augmentation procedures for the rehabilitation of deficient edentulous ridges with oral implants. Clin Oral Implants Res. 2006;17(Suppl 2):136–59. 16968389 10.1111/j.1600-0501.2006.01357.x

[5] Tan WL, et al. A systematic review of post-extractional alveolar hard and soft tissue dimensional changes in humans. Clin Oral Implants Res. 2012;23(Suppl 5):1–21. 22211303 10.1111/j.1600-0501.2011.02375.x

[6] Chiapasco M, Casentini P. Horizontal bone-augmentation procedures in implant dentistry: prosthetically guided regeneration. Periodontol 2000. 2018;77(1):213–40. 29478251 10.1111/prd.12219

[7] Buser D, Martin W, Belser UC. Optimizing esthetics for implant restorations in the anterior maxilla: anatomic and surgical considerations. Int J Oral Maxillofac Implants. 2004;19(Suppl):43–61. 15635945

[8] Dahlin C, et al. Healing of bone defects by guided tissue regeneration. Plast Reconstr Surg. 1988;81(5):672–6. 3362985 10.1097/00006534-198805000-00004

[9] Cordaro L, Amade DS, Cordaro M. Clinical results of alveolar ridge augmentation with mandibular block bone grafts in partially edentulous patients prior to implant placement. Clin Oral Implants Res. 2002;13(1):103–11. 12005140 10.1034/j.1600-0501.2002.130113.x

[10] Marx RE, et al. Severely resorbed mandible: predictable reconstruction with soft tissue matrix expansion (tent pole) grafts. J Oral Maxillofac Surg. 2002;60(8):878–88. 12149731 10.1053/joms.2002.33856

[11] Gaggl A, Schultes G, Karcher H. Distraction implants: a new operative technique for alveolar ridge augmentation. J Craniomaxillofac Surg. 1999;27(4):214–21. 10626254 10.1016/s1010-5182(99)80032-8

[12] Simion M, Baldoni M, Zaffe D. Jawbone enlargement using immediate implant placement associated with a split-crest technique and guided tissue regeneration. Int J Periodontics Restor Dent. 1992;12(6):462–73. 1298734

[13] McAllister BS, Haghighat K. Bone augmentation techniques. J Periodontol. 2007;78(3):377–96. 17335361 10.1902/jop.2007.060048

[14] Misch CE, Dietsh F. Bone-grafting materials in implant dentistry. Implant Dent. 1993;2(3):158–67. 8142935 10.1097/00008505-199309000-00003

[15] Kumar P, Vinitha B, Fathima G. Bone grafts in dentistry. J Pharm Bioallied Sci. 2013;5(Suppl 1):S125–7. 23946565 10.4103/0975-7406.113312 PMC3722694

[16] Titsinides S, Agrogiannis G, Karatzas T. Bone grafting materials in dentoalveolar reconstruction: a comprehensive review. Jpn Dent Sci Rev. 2019;55(1):26–32. 30733842 10.1016/j.jdsr.2018.09.003 PMC6354279

[17] Meloni SM, et al. Horizontal ridge augmentation using GBR with a native collagen membrane and 1:1 ratio of particulate xenograft and autologous bone: a 3 year after final loading prospective clinical study. Clin Implant Dent Relat Res. 2019;21(4):669–77. 31286654 10.1111/cid.12808

[18] Benic GI, Hammerle CH. Horizontal bone augmentation by means of guided bone regeneration. Periodontol 2000. 2014;66(1):13–40. 25123759 10.1111/prd.12039

[19] Helal MH, et al. The effectiveness of hyaluronic acid on prefabricated CAD CAM bone blocks for ridge augmentation: a split mouth study. Clin Implant Dent Relat Res. 2025;27(2):e70035. 40231349 10.1111/cid.70035

[20] Helal MH, et al. Prefabricated CAD-CAM scaffolds for management of oro-antral communication: a case report and histological analysis. Clin Implant Dent Relat Res. 2024;26(2):258–65. 38225873 10.1111/cid.13300

[21] Helal MH, et al. Osteogenesis ability of CAD-CAM biodegradable polylactic acid scaffolds for reconstruction of jaw defects. J Prosthet Dent. 2019;121(1):118–23. 29961633 10.1016/j.prosdent.2018.03.033

[22] Retzepi M, Donos N. Guided Bone Regeneration: biological principle and therapeutic applications. Clin Oral Implants Res. 2010;21(6):567–76. 20666785 10.1111/j.1600-0501.2010.01922.x

[23] Hammerle CH, Jung RE. Bone augmentation by means of barrier membranes. Periodontol 2000. 2003;33(1):36–53. 12950840 10.1046/j.0906-6713.2003.03304.x

[24] Buser D, et al. Early implant placement following single-tooth extraction in the esthetic zone: biologic rationale and surgical procedures. Int J Periodontics Restor Dent. 2008;28(5):441–51. 18990995

[25] Chappuis V, et al. Effectiveness of contour augmentation with guided bone regeneration: 10 year results. J Dent Res. 2018;97(3):266–74. 29073362 10.1177/0022034517737755

[26] Solomon S-M, et al. Finding the perfect membrane: current knowledge on barrier membranes in regenerative procedures: a descriptive review. Appl Sci. 2022;12(3):1042–62.

[27] Mandelli F, Traini T, Ghensi P. Customized-3D zirconia barriers for guided bone regeneration (GBR): clinical and histological findings from a proof-of-concept case series. J Dent. 2021;114:103780. 34400253 10.1016/j.jdent.2021.103780

[28] Arca S, et al. Personalized bone regeneration with a novel zirconia membrane: a case report. Int J Periodontics Restor Dent. 2021;41(3):391–5. 10.11607/prd.4658 36626360

[29] Schulz KF, Grimes DA. Generation of allocation sequences in randomised trials: chance, not choice. Lancet. 2002;359(9305):515–9. 11853818 10.1016/S0140-6736(02)07683-3

[30] Faul F, et al. G*Power 3: a flexible statistical power analysis program for the social, behavioral, and biomedical sciences. Behav Res Methods. 2007;39(2):175–91. 17695343 10.3758/bf03193146

[31] Wang M, et al. Clinical and radiographic outcomes of customized allogeneic bone block versus autogenous bone block for ridge augmentation: 6 Month results of a randomized controlled clinical trial. J Clin Periodontol. 2023;50(1):22–35. 36054285 10.1111/jcpe.13714

[32] Walters SJ, et al. Sample size estimation for randomised controlled trials with repeated assessment of patient-reported outcomes: what correlation between baseline and follow-up outcomes should we assume? Trials. 2019;20(1):566. 31519202 10.1186/s13063-019-3671-2 PMC6743178

[33] Charan J, Biswas T. How to calculate sample size for different study designs in medical research? Indian J Psychol Med. 2013;35(2):121–6. 24049221 10.4103/0253-7176.116232 PMC3775042

[34] Pannucci CJ, Wilkins EG. Identifying and avoiding bias in research. Plast Reconstr Surg. 2010;126(2):619–25. 20679844 10.1097/PRS.0b013e3181de24bc PMC2917255

[35] Elian N, et al. A simplified socket classification and repair technique. Pract Proced Aesthet Dent. 2007;19(2):99–104. quiz 106. 17491484

[36] Ku JK, et al. Analysis of sagittal position changes of the condyle after mandibular setback surgery across the four different types of plating systems. J Craniofac Surg. 2021;32(7):2441–5. 33710053 10.1097/SCS.0000000000007578 PMC8478309

[37] Huang LH, Neiva RE, Wang HL. Factors affecting the outcomes of coronally advanced flap root coverage procedure. J Periodontol. 2005;76(10):1729–34. 16253095 10.1902/jop.2005.76.10.1729

[38] Wu X, et al. Patient-reported outcome measures following surgeries in implant dentistry and associated factors: a cross-sectional study. BMJ Open. 2022;12(6):e059730. 35710257 10.1136/bmjopen-2021-059730 PMC9207936

[39] Corp. IBM. IBM SPSS Statistics for Windows, Version 25.0. IBM Corp.: Armonk, NY; 2017.

[40] Snecdecor GW, Cochran WG. Statistical Methods. Wiley; 1991.

[41] Abrams H, Kopczyk RA, Kaplan AL. Incidence of anterior ridge deformities in partially edentulous patients. J Prosthet Dent. 1987;57(2):191–4. 3470510 10.1016/0022-3913(87)90145-4

[42] Belser UC, et al. Aesthetic implant restorations in partially edentulous patients–a critical appraisal. Periodontol 2000. 1998;17(1):132–50. 10337321 10.1111/j.1600-0757.1998.tb00131.x

[43] Brugnami F, Caleffi C. Prosthetically driven implant placement. How to achieve the appropriate implant site development. Keio J Med. 2005;54(4):172–8. 16452826 10.2302/kjm.54.172

[44] Chappuis V, Araujo MG, Buser D. Clinical relevance of dimensional bone and soft tissue alterations post-extraction in esthetic sites. Periodontol 2000. 2017;73(1):73–83. 28000281 10.1111/prd.12167

[45] Araujo MG, et al. Alveolar socket healing: what can we learn? Periodontol 2000. 2015;68(1):122–34. 25867983 10.1111/prd.12082

[46] Al Yafi F, Alchawaf B, Nelson K. What is the optimum for alveolar ridge preservation? Dent Clin North Am. 2019;63(3):399–418. 31097134 10.1016/j.cden.2019.02.007

[47] Tonetti MS, et al. Immediate versus delayed implant placement after anterior single tooth extraction: the timing randomized controlled clinical trial. J Clin Periodontol. 2017;44(2):215–24. 27978602 10.1111/jcpe.12666

[48] Chiapasco M, Zaniboni M, Boisco M. Augmentation procedures for the rehabilitation of deficient edentulous ridges with oral implants. Clin Oral Implants Res. 2006;17(Suppl 2):136–59. 16968389 10.1111/j.1600-0501.2006.01357.x

[49] Urban IA, et al. Techniques on vertical ridge augmentation: Indications and effectiveness. Periodontol 2000. 2023;93(1):153–82. 36721380 10.1111/prd.12471

[50] Buser D, et al. Guided bone regeneration in implant dentistry: Basic principle, progress over 35 years, and recent research activities. Periodontol 2000. 2023;93(1):9–25. 38194351 10.1111/prd.12539

[51] Sasaki JI, et al. Barrier membranes for tissue regeneration in dentistry. Biomater Investig Dent. 2021;8(1):54–63. 34104896 10.1080/26415275.2021.1925556 PMC8158285

[52] Meyer M. Processing of collagen based biomaterials and the resulting materials properties. Biomed Eng Online. 2019;18(1):24. 30885217 10.1186/s12938-019-0647-0 PMC6423854

[53] Wang HL, Boyapati L. PASS principles for predictable bone regeneration. Implant Dent. 2006;15(1):8–17. 16569956 10.1097/01.id.0000204762.39826.0f

[54] Chiapasco M, et al. Customized CAD/CAM titanium meshes for the guided bone regeneration of severe alveolar ridge defects: Preliminary results of a retrospective clinical study in humans. Clin Oral Implants Res. 2021;32(4):498–510. 33548069 10.1111/clr.13720

[55] Malmstrom J, et al. Guided bone regeneration using individualized ceramic sheets. Int J Oral Maxillofac Surg. 2016;45(10):1246–52. 27364369 10.1016/j.ijom.2016.06.005

[56] Gu C, et al. Titanium mesh exposure in guided bone regeneration procedures: a systematic review and meta-analysis. Int J Oral Maxillofac Implants. 2022;37(1):e29–40. 35235627 10.11607/jomi.9098

[57] Rakhmatia YD, et al. Current barrier membranes: titanium mesh and other membranes for guided bone regeneration in dental applications. J Prosthodont Res. 2013;57(1):3–14. 23347794 10.1016/j.jpor.2012.12.001

[58] Bassir SH, et al. Outcome of early dental implant placement versus other dental implant placement protocols: a systematic review and meta-analysis. J Periodontol. 2019;90(5):493–506. 30395355 10.1002/JPER.18-0338 PMC6500770

[59] Saulacic N, et al. Impact of bone graft harvesting techniques on bone formation and graft resorption: a histomorphometric study in the mandibles of minipigs. Clin Oral Implants Res. 2015;26(4):383–91. 24547966 10.1111/clr.12357

[60] Tamilmahan P, et al. Decellularized xenogenic bone graft for repair of segmental bone defect in rabbits. Iran J Vet Res. 2022;23(4):310–21. 36874186 10.22099/IJVR.2022.40785.5906 PMC9984145

[61] Sanz M, et al. Biomaterials and regenerative technologies used in bone regeneration in the craniomaxillofacial region: consensus report of group 2 of the 15th European workshop on periodontology on bone regeneration. J Clin Periodontol. 2019;46(Suppl 21):82–91. 31215114 10.1111/jcpe.13123

[62] Galindo-Moreno P, et al. Effect of an inorganic bovine bone to autogenous cortical bone ratio upon bone remodeling patterns following maxillary sinus augmentation. Clin Oral Implants Res. 2011;22(8):857–64. 21244500 10.1111/j.1600-0501.2010.02073.x

[63] Sakr MI, et al. Early postoperative evaluation of an open-source digital workflow for designing custom-made zirconia membranes in maxillary guided bone regeneration. BMC Oral Health. 2025;25(1):1200. 40682045 10.1186/s12903-025-06592-0 PMC12275381

[64] Weissheimer A, et al. Fast three-dimensional superimposition of cone beam computed tomography for orthopaedics and orthognathic surgery evaluation. Int J Oral Maxillofac Surg. 2015;44(9):1188–96. 25935632 10.1016/j.ijom.2015.04.001 PMC4526318

[65] Gultekin BA, et al. Comparison of bone resorption rates after intraoral block bone and guided bone regeneration augmentation for the reconstruction of horizontally deficient maxillary alveolar ridges. Biomed Res Int. 2016;2016(1):4987437. 27847815 10.1155/2016/4987437 PMC5101362

[66] Yu SH, Saleh MHA, Wang HL. Simultaneous or staged lateral ridge augmentation: a clinical guideline on the decision-making process. Periodontol 2000. 2023;93(1):107–28. 37529966 10.1111/prd.12512

[67] Saravanan P, et al. Efficacy of guided bone regeneration using composite bone graft and resorbable collagen membrane in Seibert’s Class I ridge defects: radiological evaluation. J Oral Implantol. 2013;39(4):455–62. 23964779 10.1563/AAID-JOI-D-10-00211

[68] Heikal M, Shuman M, Khalil M. Evaluation of bone regeneration for augmentation of alveolar bone using 3D computer-guided ceramic Sheets. Al-Azhar J Dent Sci. 2020;23(4):393–9.

[69] Albash Z et al. Horizontal augmentation using customized zirconia membrane: a case report. Open Dentistry J. 2024;18(1). 10.2174/0118742106332436240820100241

[70] Souidan A, Ayad S, Sweedan A. Augmentation of the anterior maxillary bony defects using custom-made zirconia membranes (a case series). Alexandria Dent J. 2022;0(0):0–0.

[71] Uriarte X, et al. Zirconia barriers in bone regeneration procedures: a scoping review. Clin Oral Implants Res. 2025;36(4):411–22. 39846616 10.1111/clr.14404

[72] Thoma DS, et al. Efficacy of lateral bone augmentation performed simultaneously with dental implant placement: a systematic review and meta-analysis. J Clin Periodontol. 2019;46(Suppl 21):257–76. 30675733 10.1111/jcpe.13050

[73] Keddar M, Evrard L, Shall F. Horizontal ridge augmentation using guided bone regeneration with an association of particulate allografts mixed with platelet-rich fibrin, collagen membrane and tent-screws: A prospective study. J Stomatol Oral Maxillofac Surg. 2024;125(12 Suppl 2):101872. 38582352 10.1016/j.jormas.2024.101872

[74] Nabil Melek L. Comparison of two pre-prosthetic surgical techniques for augmentation of mandibular vertical ridge defects. J Osseointegration. 2021;14(1):59–63.

[75] Urban IA, et al. Long-term evaluation of peri-implant bone level after reconstruction of severely atrophic edentulous maxilla via vertical and horizontal guided bone regeneration in combination with sinus augmentation: a case series with 1 to 15 years of loading. Clin Implant Dent Relat Res. 2017;19(1):46–55. 27238406 10.1111/cid.12431

[76] Collins SL, Moore RA, McQuay HJ. The visual analogue pain intensity scale: what is moderate pain in millimetres? Pain. 1997;72(1–2):95–7. 9272792 10.1016/s0304-3959(97)00005-5

[77] Urban T, Wenzel A. Discomfort experienced after immediate implant placement associated with three different regenerative techniques. Clin Oral Implants Res. 2010;21(11):1271–7. 20528891 10.1111/j.1600-0501.2010.01943.x

[78] Salahi S, et al. Patient-reported outcomes in intraoral bone block augmentation compared to gbr procedures before implant placement: a systematic review. J Clin Med. 2025;14(15):5331. 40806953 10.3390/jcm14155331 PMC12347253

[79] Kofina V, et al. Patient-reported outcomes following guided bone regeneration: correlation with clinical parameters. J Dent. 2023;136:104605. 37419383 10.1016/j.jdent.2023.104605

[80] Dawar A, et al. Short-term patient-reported outcomes correlate with clinical parameters in guided bone regeneration. Evid Based Dent. 2025;26(1):32–3. 39939358 10.1038/s41432-025-01115-w

